# Assessing ChatGPT’s orthopedic in-service training exam performance and applicability in the field

**DOI:** 10.1186/s13018-023-04467-0

**Published:** 2024-01-03

**Authors:** Neil Jain, Caleb Gottlich, John Fisher, Dominic Campano, Travis Winston

**Affiliations:** Department of Orthopedic Surgery, Texas Tech University Health Sciences Center Lubbock, 3601 4th St, Lubbock, TX 79430 USA

**Keywords:** ChatGPT, OITE, Resident Education, General Orthopedics, Machine Learning

## Abstract

**Background:**

ChatGPT has gained widespread attention for its ability to understand and provide human-like responses to inputs. However, few works have focused on its use in Orthopedics. This study assessed ChatGPT’s performance on the Orthopedic In-Service Training Exam (OITE) and evaluated its decision-making process to determine whether adoption as a resource in the field is practical.

**Methods:**

ChatGPT’s performance on three OITE exams was evaluated through inputting multiple choice questions. Questions were classified by their orthopedic subject area. Yearly, OITE technical reports were used to gauge scores against resident physicians. ChatGPT’s rationales were compared with testmaker explanations using six different groups denoting answer accuracy and logic consistency. Variables were analyzed using contingency table construction and Chi-squared analyses.

**Results:**

Of 635 questions, 360 were useable as inputs (56.7%). ChatGPT-3.5 scored 55.8%, 47.7%, and 54% for the years 2020, 2021, and 2022, respectively. Of 190 correct outputs, 179 provided a consistent logic (94.2%). Of 170 incorrect outputs, 133 provided an inconsistent logic (78.2%). Significant associations were found between test topic and correct answer (*p* = 0.011), and type of logic used and tested topic (*p* =  < 0.001). Basic Science and Sports had adjusted residuals greater than 1.96. Basic Science and correct, no logic; Basic Science and incorrect, inconsistent logic; Sports and correct, no logic; and Sports and incorrect, inconsistent logic; had adjusted residuals greater than 1.96.

**Conclusions:**

Based on annual OITE technical reports for resident physicians, ChatGPT-3.5 performed around the PGY-1 level. When answering correctly, it displayed congruent reasoning with testmakers. When answering incorrectly, it exhibited some understanding of the correct answer. It outperformed in Basic Science and Sports, likely due to its ability to output rote facts. These findings suggest that it lacks the fundamental capabilities to be a comprehensive tool in Orthopedic Surgery in its current form.

*Level of Evidence*: II.

## Introduction

Large language models (LLM) are learning models designed to understand and output natural language [1]. LLM’s are built on Transformer, a neural network architecture that uses a self-attention mechanism to achieve better understanding of input data [1, 2]. These models have recently gained widespread mainstream media attention via the release of Chat Generative Pre-trained Transformer, also popularly known as ChatGPT [3]. ChatGPT is a conversational chatbot released in November 2022 by OpenAI, a San Francisco-based research and deployment company whose declared mission is to ensure that artificial general intelligence benefits humanity [1, 4]. It uses its training on GPT 3.5, a LLM with at least 175 billion parameters, and 570 gigabytes worth of information from books, articles, and websites, to generate natural human-like responses to input prompts [1, 3, 4].

ChatGPT builds on previous GPT 3.5 models with the addition of a reinforcement learning technique so that users can continuously offer feedback to shape its behavior [1, 3]. As a result of these improvements, it represents a pinnacle of human achievement in the field of artificial intelligence. It has shown its prowess in medicine by passing parts of the United States Medical Licensing Exam, offering recommendations for breast cancer screening and prevention, and demonstrating a broad range of knowledge in fields such as Obstetrics/Gynecology or Gastroenterology/Hepatology [3, 5–8].

In this study, we sought to examine ChatGPT’s performance on the Orthopedic In-Service Training Exam (OITE) that resident physicians take yearly. This exam, first introduced in 1963 by the American Academy of Orthopedic Surgeons, is highly regulated and standardized nationwide to cover a broad range of 11 topics in Orthopedics [9]. Its difficulty and complexity have established it as an excellent benchmark for residents to assess their knowledge and competence compared to their peers in the same year [9]. The feedback it provides allows test takers to understand what areas may need improvement before they take the American Board of Orthopedic Surgery part 1 examination [9]. Our work analyzed ChatGPT’s test results and rationales both qualitative and quantitatively to see if widespread adoption as a resource in the field is feasible.

## Methods

After completion of a literature search using the PubMed and Google Scholar databases, it was found that limited literature exists focusing on the application of ChatGPT in the realm of Orthopedic Surgery. This study sought to employ a modified methodology developed by Kung et al. to objectively assess ChatGPT’s performance on a standardized test [5]. The free May 3rd, 2023 version of ChatGPT-3.5 was used for all parts of this project. No subscription was made to the ChatGPT Plus model which runs on GPT-4, a more advanced LLM that extends past the year 2021. Old OITE exam questions from the years 2020, 2021, 2022 were obtained and collaboratively screened by two primary authors (NJ, CG). Specific inclusion criteria included all questions with four multiple choice answers and text only. Due to ChatGPT’s limitations, exclusion criteria included all questions with images, photography, or tables. Because ChatGPT uses memory retention to improve its performance, a new chatbox was created each time a question was input to reduce bias [5].

Analysis of the data set was performed to initially look at how many questions ChatGPT-3.5 correctly answered. These results were analyzed against the OITE Technical Reports put out yearly to assess ChatGPT’s performance compared to resident physicians. Questions were typed according to the one domain of the OITE that they most closely aligned with. A primary evaluator (NJ, PGY-1) listed possible domains each question could identify with. A second senior evaluator (CG, PGY-4) chose a single domain each question most closely aligned with. If a domain could not be picked after two evaluators, a fellowship trained board-certified orthopedic surgeon (TW) made a final decision. To assess the decision-making process used by ChatGPT, its generated rationales were classified according to their consistency with the explanations provided by the authors of the OITE. This was achieved by creating 6 groups as follows:

- CC—correct answer, consistent logic.
- CIC—correct answer, inconsistent logic.
- IC—incorrect answer, consistent logic.
- IIC—incorrect answer, inconsistent logic.
- CN—correct answer, no logic provided.
- IN—incorrect answer, no logic provided.

These variables were collected and analyzed using contingency table construction and Chi-squared analyses. Statistical analysis was performed using IBM SPSS Statistics software (IBM Corp. Released 2022. IBM SPSS Statistics for Windows, Version 29.0. Armonk, NY: IBM Corp).

## Results

Out of a total of 635 questions, 360 were able to be used as inputs in this study (56.7%). ChatGPT scored 55.8%, 47.7%, and 54% for the 2020, 2021, and 2022 OITEs, respectively (Table 1). Among accredited resident physicians, this corresponded to performance between a PGY-1 and PGY-2, below a PGY-1, and at the level of a PGY-1, for the years 2020, 2021, and 2022, respectively.

**Table 1:** Total questions answered correct or incorrect categorized by year

	Year			
	2020	2021	2022	Total
Correct				
No	57	56	57	170
Yes	72	51	67	190
Total	129	107	124	360

**Fig. 1:**
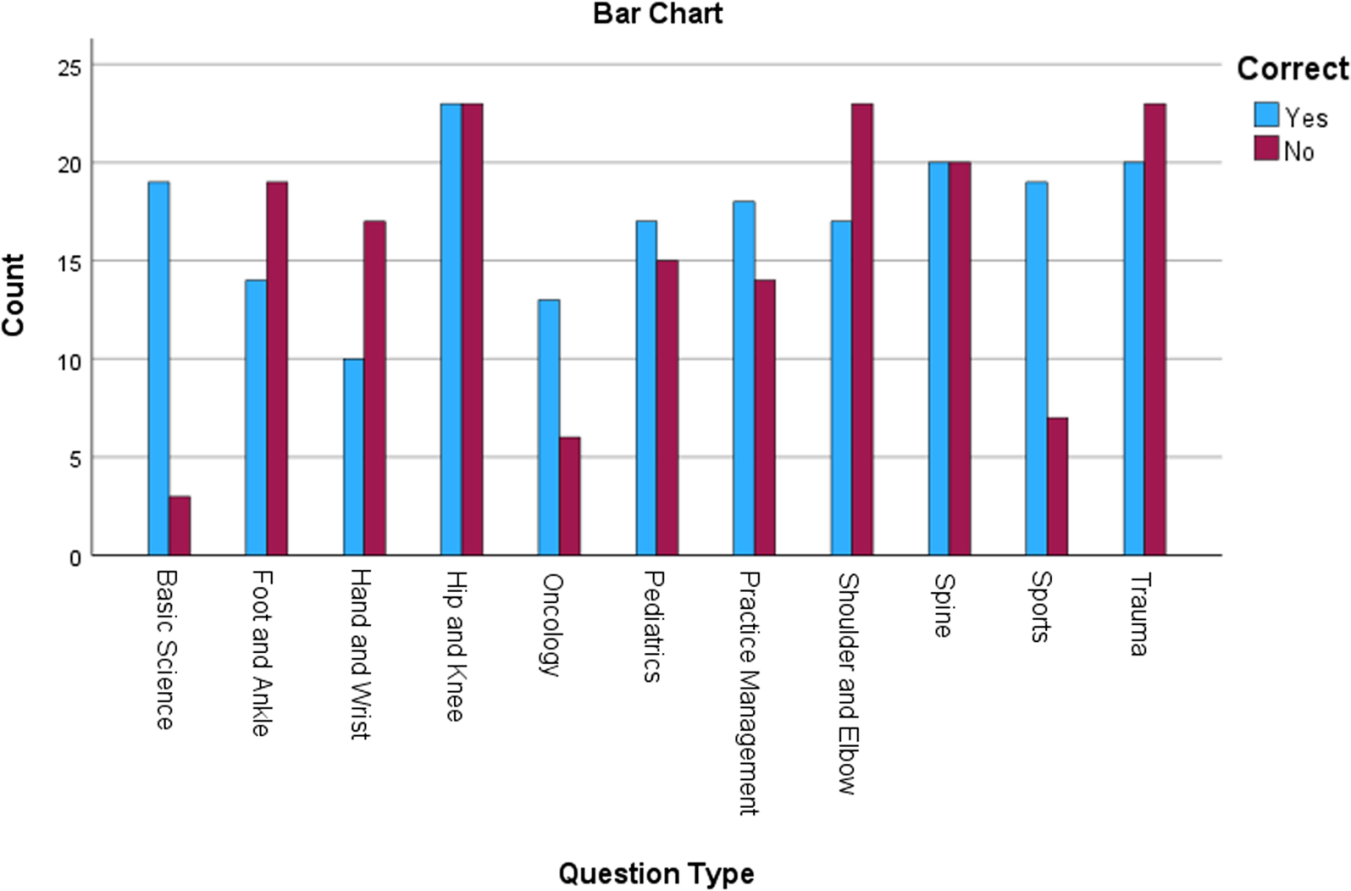
Correct or incorrect answers categorized by subject type

**Fig. 2:**
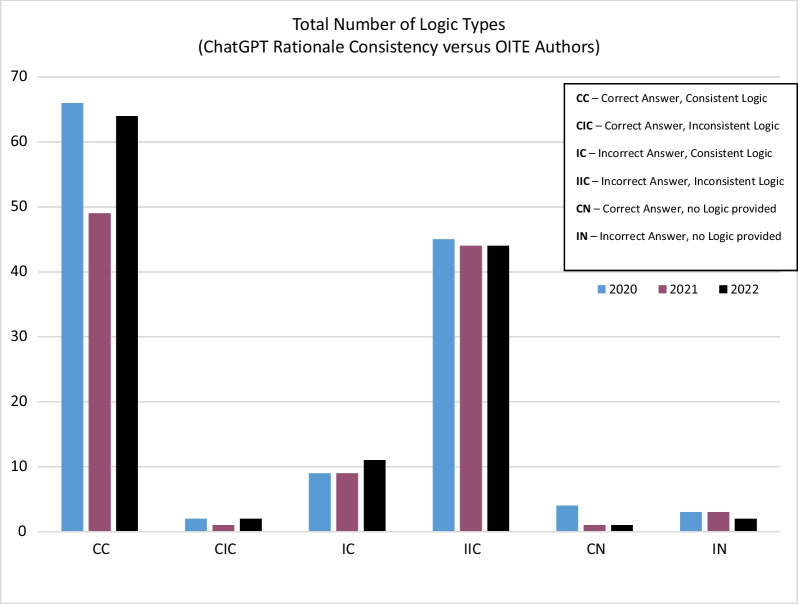
Total number of logic types by year

Total questions correctly answered by topic, and total number of questions included in each topic are shown in Table 2 and Fig. 1. Total number of logic types by year are shown in Fig. 2. Question type and logic group distribution are shown in Table 3.

**Table 2:** Correct or incorrect answers categorized by subject type

	Correct		
	No	Yes	Total
Question Subject Type			
Basic Science	3	19	22
Foot and Ankle	19	14	33
Hand and Wrist	17	10	27
Hip and Knee	23	23	46
Oncology	6	13	19
Pediatrics	15	17	32
Practice Management	14	18	32
Shoulder and Elbow	23	17	40
Spine	20	20	40
Sports	7	19	26
Trauma	23	20	43
Total	170	190	360

**Table 3:** Question type and logic group distribution

	CC	CIC	CN	IC	IIC	IN	Total
Question Type							
Basic Science	14	0	5	1	2	0	22
Foot and Ankle	14	0	0	3	15	1	33
Hand and Wrist	9	1	0	2	14	1	27
Hip and Knee	23	0	0	3	19	1	46
Oncology	12	1	0	2	4	0	19
Pediatrics	16	1	0	3	12	0	32
Practice Management	18	0	0	1	12	1	32
Shoulder and Elbow	17	0	0	4	17	2	40
Spine	18	1	1	5	14	1	40
Sports	18	1	0	3	4	0	26
Trauma	20	0	0	2	20	1	43
Total	179	5	6	29	133	8	360

Question Type * Logic (CC = correct consistent, CIC = correct inconsistent, IC = incorrect consistent, IIC = incorrect, inconsistent, CN = correct, no reasoning, IN = incorrect, no reasoning) Crosstabulation

Of the 190 correct outputs, 179 provided a true consistent (CC) logic in answering the question (94.2%). Of the 170 incorrect outputs, 133 provided a true inconsistent (IIC) logic in answering the question (78.2%).

A Chi-squared test was conducted to examine the associations between question topic, year administered, and whether the question was answered correctly. The test revealed a significant association with test topic and correct answer (*χ*^2^ (10) = 23.020, *p* = 0.011). Post hoc analysis revealed that the Basic Science and Sports categories had adjusted residuals greater than 1.96, indicating a statistically significant departure from the expected frequencies. No significant association with test year and correct answer was observed (χ^2^ (2) = 1.678, *p* = 0.432.).

A Chi-squared test was used to explore an association between the type of logic used and tested topic. The test resulted in a significant association (χ^2^ (50) = 99.386, *p* =  < 0.001). Post hoc analysis showed that Basic Science and correct, no logic (CN); Basic Science and incorrect, inconsistent logic (IIC); Sports and correct, no logic (CN); and Sports and incorrect, inconsistent logic (IIC); had standard adjusted residuals greater than 1.96, indicating a statistically significant departure from the expected frequencies.

## Discussion

Computer algorithms that employ large datasets to imitate how humans use experience to improve decision making defines a subset of artificial intelligence known as machine learning (ML) [10]. The potential of machine learning in medicine is that it can automate tasks, assist in provider thought processes, and improve perioperative treatment management [10, 11]. In the field of Orthopedic Surgery, machine learning has already enabled detection of fractures, loose implants, or even helped diagnose tumors [10].

### Limitations of ChatGPT

ChatGPT represents a milestone in ML in that it builds on previous GPT 3.5 models with the addition of a reinforcement learning technique so that users can continuously offer feedback to shape its behavior [1, 3]. However despite this, it has important restrictions worth considering. Its training on GPT 3.5 is limited in scope until the end of 2021, with it having inadequate knowledge of events beyond that time frame [1]. This has often led to it having a tendency to fabricate references or have incorrect reasoning when solving problems that require logic beyond this date [1, 12–14]. Kung et al. found that GPT-3.5 used verifiable sources for answering OITE questions in 47.2% of its outputs but did not provide a detailed breakdown based on response type or logic used [15]. Our study’s assessment found that ChatGPT primarily demonstrated consistent logic with testmakers when answering correctly, and inconsistent logic when answering incorrectly. A detailed categorization into how often fabricated references are used in the context of CC versus IIC logic groups is needed. This would provide a deeper technical assessment into whether ChatGPT fabricates more often for correct versus incorrect answers.

Another important limitation is that the standard version of ChatGPT (GPT-3.5) used in this report cannot interpret inputs with visual aids [3, 5]. Diagnosing musculoskeletal disorders or managing perioperative patients both rely on the use of interval imaging. Upgrading to ChatGPT Plus (GPT-4) would have allowed visual inputs; however, these capabilities continue to be benchmarked, refined, and limited [16, 17]. At first glance, ChatGPT-3.5’s inability to provide outputs in these situations makes it seem impractical in the field. However, studies have attempted to find other applicable uses for it. Dubin et al. replicated a patient’s online search about arthroplasty procedures to assess whether ChatGPT provided more appropriate resources against a popular online search engine [10]. Their results had heterogeneous responses between the two groups with ChatGPT more commonly using higher quality websites such as PubMed or the American Academy of Orthopedic Surgeons for its information [10]. They suggested that it shows promise as a valuable resource for patient education [10]. Studies done in cardiovascular disease prevention and breast cancer screening have made similar recommendations [6]. Dubin and colleagues also called for further work into whether it can be used to augment the surgical consenting process to improve patient education [10]. A randomized controlled trial found that using an online educational resource in the consent process for orthopedic elective surgeries leads to significant increases in patient knowledge and satisfaction [10, 18]. The authors used an online website as their resource, however ChatGPT could potentially fill this role and be used to answer frequently asked questions or explain concepts. Overall, LLM’s such as ChatGPT represent an evolving technology and models including specific training on clinical data sets may change these recommendations.

### Performance on the United States Medical Licensing Exam (USMLE)

Kung et al. examined ChatGPT’s performance on all three parts of the USMLE using publicly available questions directly from the official website [5]. The formats were altered such that some were open ended, while others remained multiple choice either requiring justification or not [5]. The authors found ChatGPT scored the lowest on Step 1 material amongst all three question types when indeterminate answers were included [5]. As Step 1 is largely basic science and pathology based, these results contrast with observations seen in our study. The OITE covers 11 domains of Orthopedics and incorporates radiographs and current treatment standards for common pathophysiology into its question sets [9]. ChatGPT answered 19/22 (86.4%) of Orthopedic Basic Science questions correct with 1 out of 3 incorrect responses still demonstrating consistent logic with the testmakers (IC group). In comparison, the second subsequently high performing subject area was Sports Medicine with only 18/26 (69.2%) correct. Based on our findings, it appears that ChatGPT demonstrates strength in answering questions that do not require a true open-ended logic processing. In the Basic Science questions presented, searching its trained data and outputting an answer was typically enough for ChatGPT to respond correctly with little additional reasoning needed.

In their examination of internal concordance between input and output, Kung et al. found the rate among accurate responses was 99% in comparison to 85% for incorrect answers.[5] Our study utilized a different approach and examined the logic used in comparison to OITE testmakers. In a similar fashion, we found that when answering correctly, ChatGPT provided logic that was consistent with the testmakers (CC); however, this rate dropped by 16% when answering incorrectly (IIC). This seems to suggest that when answering correctly, ChatGPT displayed confidence in its responses and was able to pinpoint a more exact logic amongst its training data. However, it faltered when answering incorrectly and other logic groups, such as having an incorrect answer with consistent logic (IC), saw increases. In emulating a test taker, ChatGPT may be demonstrating some understanding of the correct answer but fails to make an accurate guess.

Using 60% as a passing threshold, Gilson et al. tested different question sets and found that ChatGPT was capable of answering Step 1 and Step 2 questions correctly at rates higher than this standard [3]. Their results indicated that ChatGPT performs at a level expected of a third-year medical student when medical knowledge is assessed [3]. Greater than 90% of the answers they received included a rationale, whether right or wrong [3]. They observed that correct answers more often pulled information external to the question, suggesting again that ChatGPT’s abilities to answer questions are restricted to whether it can relate input to data found within its training dates [3]. Paradoxically, our study did not support this. We found that ChatGPT’s performance averaged around an accredited first year orthopedic resident physician. On the 2021 OITE it dipped by approximately seven percent, but then rebounded back to 54% on the following year. We had hypothesized that due to new orthopedic treatment options being introduced yearly, ChatGPT would perform worse on the 2022 examination, a year past its training cutoff. However, one possible reason may be the variation in question visual aid distribution year to year. 2022 returned to 13 usable Sports questions, similar to the 10 in 2020; whereas 2021 only had 3. Sports and Basic Science were noted to be answered at higher correct answer rates in comparison to other subjects, suggesting that the addition of these strong areas as inputs for ChatGPT led to a higher percentage score.

### ChatGPT in other fields

In taking the American Heart Association’s Basic Life Support and Advanced Cardiovascular Life Support examinations, ChatGPT did not reach a passing threshold. However, researchers noted that the answers provided by ChatGPT did not simply answer the question but also provided insightful explanations, regardless of whether the answer was correct or incorrect [19]. This was further seen in a query project done in the field of Obstetrics and Gynecology by Grünebaum et al. [7] Although its answers were mostly on target, its responses to queries were “nuanced, eloquent, informed, and had virtually no grammatical errors" [7]. We also found that despite no prompting by the input, ChatGPT provided explanations as to how it arrived at its answer choice. Only 14 out of 360 questions provided no logic (CN, IN), comprising roughly 3.9% of the dataset. Of these, nearly a third came from Basic Science (5/14), likely due to ChatGPT being an AI model that excels in producing rote facts.

In Korea, ChatGPT performed worse than medical students on a parasitology exam [20]. As noted by the author, a reason for this may be due to a lack of knowledge about Korea’s unique epidemiologic data by ChatGPT. This data is not searchable or is available only in Korean [20]. Similar limitations were reported by Yeo et al., in which they noted that ChatGPT was unable to identify specific cutoffs for the management of cirrhosis or make guideline recommendations for hepatocellular carcinoma screening [8]. They highlighted that this is likely due to guidelines varying regionally or between countries [8]. In the realm of Orthopedics, this may translate to ChatGPT being unable to offer comprehensive treatment recommendations outside of demonstrating general basic knowledge. Operative handling for injuries may vary between institutions and ChatGPT may promote direct contradictions to recommendations made by the treating surgeon. Further refinement of its training set is required in order to fine tune its responses so that individuals may receive more personalized recommendations based on geographic location.

### Teaching applicability

Several studies have examined the teaching applicability of ChatGPT in medicine. Kung et al. used adjucators to examine the responses set forth by ChatGPT based on the criteria of “novelty, obviousness, and validity" [5]. They found that in its responses, ChatGPT produced at least one new insight 88.9% of the time [5]. When normalizing the number of insights against possible answer choices, they found that the average density of insights was higher in questions answered correctly versus incorrectly [5]. This suggests that comparatively there is value in learning from ChatGPT’s correct answers. However, this becomes problematic as its correct answer rate on usable Orthopedic questions ranged from 47.7 to 55.8%. In the areas of Basic Sciences and Sports, both were answered correctly at higher rates than other topics. Post hoc analysis found that these used correct answer with no logic (CN) and incorrect answer with inconsistent logic (IIC) at higher frequencies than other pairings. These results indicate that ChatGPT may be mimicking the cognitive processing of test taking. In these situations, individuals either answer correctly with a firm resolve, or get stuck on questions and must develop a rationale for an incorrect answer or educated guess. Further work to improve the knowledge and reasoning level of ChatGPT is needed in order to have it answer accurately at increased rates. While it offers great explanations, it appears difficult for a student or resident physician to learn from it if the rationale may be wrong approximately half the time.

Other suggestions have been made for ChatGPT’s educational applicability through use in small group settings [3, 11]. Due to its ability to provide human like dialogic responses, it could fill the role of a peer to enable individuals studying independently to ask for clarification on hard to understand medical concepts, diagnoses, or treatments [3]. A meta-analysis on this form of teaching showed that peer learning is as efficacious as learning from faculty [3, 21]. Multiple studies have commented that the quality of ChatGPT’s explanations are of high clarity and relevance with low levels of self-contradiction [5, 8, 19, 22].

### Limitations of the study

A large percentage of cases reviewed by orthopedic surgeons require imaging in creating differential diagnoses. As noted previously, the inability of ChatGPT-3.5 to interpret inputs with visual aids cannot be overlooked. This led to a large portion of the available questions (43.3%) being unusable and limited the study to assessing ChatGPT-3.5’s logic when visual inputs were excluded. In doing so, there was an inherent burden placed on it to perform at a high level so that future AI iterations could build on its logical processing and accept radiographic inputs. While it was observed that the OITE typically aims for half the test to have imaging, there was an uneven distribution noted for the 2021 OITE exam. It had more questions with visual aids, and this led to a decrease in power by approximately 17–22 usable questions in comparison to other test years used in this study. Another notable limitation in this study regarded ChatGPT-3.5’s limited knowledge of events past the end of 2021. Standards of care in Orthopedics are constantly evolving and ChatGPT is a resource that cannot access the internet. In the data set, we received 6 indeterminate responses—4 in 2020, and 2 in 2022. These responses were all counted as incorrect, inconsistent logic (IIC) as we believed they represented a test taker leaving a question blank on an examination. Removing these questions from the data set may skew the results of the present study. Third, ChatGPT receives regular updates to its interface and likely improvements to its logic processing as well. This study used the May 3rd, 2023 version of ChatGPT-3.5, which was soon outdated after data collection. LLM’s represent an evolving technology, and the present study reported on ChatGPT’s OITE performance using the most available version at the time. The efficacy of future iterations of ChatGPT-3.5 warrant further investigation. Fourth, the free version of ChatGPT (GPT-3.5) was utilized in the entirety of this study. ChatGPT Plus, a paid subscription model running on GPT-4, can receive visual inputs and has knowledge of events well beyond its free counterpart. Further studies corroborating it in orthopedic surgery are needed. Fifth, questions used as inputs were categorized into only one domain of Orthopedics that they most closely identified with. During the classification process, it was observed that some questions potentially covered parts of multiples domains and it was left up to the authors’ discretion as to which domain it most closely assessed. Misclassification of multiple questions may have affected conclusions of ChatGPT’s performance across domains, but would not have changed logic analysis. Lastly, this study offers only a small insight into what to expect from ChatGPT in the field of Orthopedics. To fully assess its competency, it must be challenged in real life situations to assess its performance amongst students, residents, or practicing surgeons.

## Conclusion

The primary findings of this study indicate that ChatGPT-3.5 (May 2023) answered OITE questions correctly approximately half the time with its performance averaging around the level of a PGY-1 resident physician. When answering correctly, it displayed congruent reasoning with testmakers. When answering incorrectly, it exhibited some understanding of the correct answer. It outperformed in Basic Science and Sports, likely due to its ability to output rote facts. These findings, ChatGPT-3.5’s (May 2023) inability to interpret radiographic inputs, and its potential inability to know regional treatment variances, suggest that it lacks the fundamental capabilities to be a comprehensive tool in Orthopedic Surgery in its current form. It may have limited applications in general patient perioperative education as it has been noted to use higher quality sources in its outputs when compared to other search engine counterparts. Given the evolving nature of artificial intelligence, the benchmarks established in this study may be used to make comparisons for future LLMs and influence model design to include more specific training on clinical data sets.

## Data Availability

The OITE questions used for this study represent copyrighted material that are not publicly available. A deidentified data sheet is available from the corresponding author upon reasonable request.
